# An open chat with… Johannes Herrmann

**DOI:** 10.1002/2211-5463.13879

**Published:** 2024-08-23

**Authors:** Ioannis Tsagakis, Johannes Herrmann

**Affiliations:** ^1^ FEBS Open Bio Editorial Office Cambridge UK; ^2^ Cell Biology University of Kaiserslautern Kaiserslautern Germany

## Abstract

Johannes Herrmann is a Professor of Cell Biology at the University of Kaiserslautern in Germany. His research focus is centred on the biogenesis of mitochondrial proteins. Johannes is a member of the German Academy of Sciences and member of the Deutsche Forschungsgemeinschaft (DFG, German Research Foundation) Biochemistry Panel. He has been a member of the FEBS Publication Committee since 2021. In this interview, he explains the source of his scientific interest in mitochondrial biology and details how the work of the FEBS Publication Committee safeguards the future of the FEBS Press journals.

## Graphical abstract

Johannes Herrmann is a Professor of Cell Biology at the University of Kaiserslautern in Germany. In this interview, he explains the source of his scientific interest in mitochondrial biology and how the work of the FEBS Publication Committee safeguards the future of the FEBS Press journals.

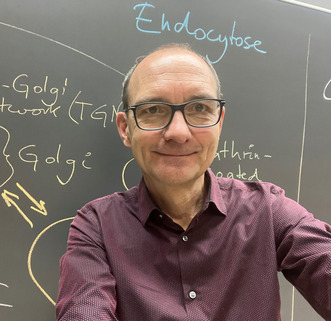

## Please tell us a bit about your career, how you got there, and why you embarked on a career in research?

I have always been a curiosity‐driven person. As a child, I drove my parents nuts during our holidays, when I woke up at 5:00 am to go bird‐watching and stroll through nature. I still go into the deep forests around our city for long hikes every free weekend I get (luckily my wife enjoys this as much as I do). I just love to discover things I have not seen before. When hiking, I regularly get lost, even on purpose, heading in new directions as I find it extremely rewarding if I suddenly bump into paths that I know. It is just so cool when things fall into place and get connected.

My career as a researcher is actually inspired by the same motivation. As a student, I particularly liked cell biology and all the molecular mechanisms in the microcosmos of a cell. I was particularly intrigued by mitochondria because they are like mini‐worlds in our cells. They have their own genome, systems for protein synthesis and folding, a complex metabolism, and a large degree of autonomy from other cellular processes. Still, they are absolutely dependent on the rest of the cell, they import most of their proteins from outside and are tightly integrated into cellular processes.

I was very fortunate as a PhD student to join the laboratory of Walter Neupert in Munich, a world‐leading hotspot of mitochondrial research. There, I studied the biogenesis of mitochondria‐encoded and imported proteins using yeast as a model. I continued working with yeast for my postdoc with Randy Schekman at UC Berkeley but changed the focus from mitochondria to the endoplasmic reticulum (ER). Today, in my laboratory, many projects address interactions of mitochondria with the ER, both being fascinating organelles. Thus, just as in our forests, I stroll around and love to explore new territory and find unexpected new connections between these cellular compartments.

## Please tell us a bit about your role at the FEBS Publication Committee for readers who are not aware, what are the type of issues you might cover at the FEBS Publication Committee? Please provide an example

The Federation of European Biochemical Societies (FEBS) is an organization with over 30 000 members from all over Europe and neighbouring regions. This includes the European Union countries as well as Turkey, Israel, etc., and also many from the East such as Georgia or Armenia. Thus, FEBS offers a wonderful platform to support interactions between biochemists and biologists from very different regions—this is a major motivation for me to be active in FEBS.

The Publication Committee discusses, supports and oversees the operation and development of the four journals of FEBS: *FEBS Letters*, *FEBS Journal*, *FEBS Openbio* and *Molecular Oncology*. The Publication Committee helps journals to attract excellent submissions from a broad authorship, to find well‐respected editors, and to have close cooperation with Wiley as their publisher. With these journals, FEBS can generate money to support the FEBS courses and meetings as well as FEBS fellowships. Thus, I think being active for these journals supports our scientific community, but I also just enjoy the meetings with other active European scientists at the meetings of the Publication Committee.

## Please fill in the blank: Mitochondria are the ____ of the cell? What are some really cool aspects of mitochondrial biology that a lot of people would not be aware of?

Mitochondria are the **hot heart** of the cell!

Mitochondria are much more than just ATP generators. They are for example important signalling platforms. Did you know that the antiviral signalling of human cells takes place on the mitochondrial surface? Thus, mitochondria are of central relevance for the defence of cells against viruses. This is why for example the SARS CoV‐2 virus induces the production of high amounts of a protein called Orf9b, which blocks a mitochondrial surface receptor to interfere with antiviral signalling. This viral protein camouflages the virus by binding to the mitochondrial Tom70 receptor and thereby also disturbs mitochondrial biogenesis [1, 2].

Another cool aspect is the ability of mitochondria to eject their genome into the cytosol to trigger inflammation and a defence programme against bacteria [3]. Eukaryotic cells can sense the presence of bacterial DNA in their cytosol, which induces a very strong defence programme. However, in the absence of bacteria, this programme can be triggered by mitochondrial DNA which is ejected into the cytosol. Since the mitochondrial genome has similar features to that of bacteria, for example an absence of histones, the release of mitochondrial DNA is a cell‐autonomous mechanism to unleash this powerful defence system.

## Looking at your own research career and interests, what are you most proud of and what keeps you awake at night?

I think the story my laboratory is currently working on is the coolest discovery we ever made! I have published almost 200 articles over the last 25 years. Still, we just found some exciting and unexpected aspects of mitochondrial biology. This is what keeps me awake at night, literally. You also asked what I am proud of. The word pride does not properly describe what I feel about our discoveries—however, I am very proud of (and grateful for) my wonderful team—we have an amazing laboratory family and I enjoy working with them every single day.

## What do you consider your most influential publication and why?

The coolest stories are those that are not told yet and which are just emerging. For example, we just discovered that mitochondria deliberately produce a highly aggregation‐prone protein that is encoded in their genome. This protein gives rise to intramitochondrial aggregates which can protect cells from misfolded proteins under extreme stress conditions. This protein serves as a nucleation site for aggregates and absorbs and sequesters other proteins. A cool mechanism and again, a completely unexpected new aspect of mitochondrial biology.

## What do you like most and least about working in research?

As I said before, I love to discover things. However, the best aspect of being a researcher is the stimulating interactions with so many interesting people. The daily discussions about experiments and results with the PhD students and postdocs of my team, teaching students in many different courses, and the interactions with colleagues at conferences and meetings—these social interactions with others are what makes the job of a professor the best profession one can think of.

What I like the least: bureaucracy and fighting with editors and referees is not an enjoyable aspect of our work.

## What changes do you think we'll see in terms of the overall research infrastructure over the next 5 to 10 years?

Wow, this is a very general question. I think there are two major developments: 1) data sets are gettting larger and larger and 2) artificial intelligence (AI). When I was a PhD student, most papers showed western blots and simple figures. Now most papers show omics data and complex data sets. The complexity will continue to increase further and soon most studies will show omics data from many single cells or tissues. This is not only extremely challenging and requires computational biologists, but also makes papers very difficult to read as they often appear unfocused and overly generalized.

The second important trend is the influence of artificial intelligence and machine learning. This offers wonderful opportunities, for example for the analysis of these large data sets, but it also makes it very difficult to identify manipulated and fraudulent studies.

## In 2010, you won an award for Excellent Teaching in Rheinland‐Pfalz. In your opinion, what impact has/does/will AI have on students' university work?

Actually, I think AI will have an immense effect on research. Alphafold is an excellent example of this potential. However, I do not see AI as being so relevant for students and teaching. Research and publications have changed a lot over the last 25 years. However, how students learn the structures of amino acids or the steps of glycolysis did not change. To be honest, my teaching is extremely old‐fashioned and traditional. For example, I like to make big drawings with chalk on a blackboard to explain the steps of mitosis or how proteins are degraded by the proteasome. This teaching style is rather traditional, but perhaps it is the reason why the students awarded me a teaching prize.

## You have an account on X (formerly *Twitter*) which you use to highlight your and others' research, and a respectable number of people follow you. What are the biggest benefits you have reaped from its use as a scientist that you would use as arguments to convince peers to jump on this ship? What should fellow scientists be wary of if they decide to engage in sci‐com through social media?

I really loved Twitter to hear about novel discoveries from the laboratories and people I followed, and to tell others about our unpublished observations. For me, Twitter was an alerting platform, and the short format of the tweets was perfect for this. Unfortunately, since it changed to X, there are advertisements and content that is not related to science. So, many scientists became less active, myself included.

## Your most influential tweet (most retweets) was about MitoStores—what's that about?

We discovered that mitochondrial precursor proteins can be transiently stored in the cytosol if their levels exceed the mitochondrial import capacity. Small heat shock proteins serve as nucleation sites to absorb these precursors, thereby forming aggregates which we called MitoStores [4]. As soon as the mitochondria ramp up their import capacity, the precursors are released from the MitoStores and imported into mitochondria. We placed a tweet with this story on Twitter (https://twitter.com/Herrmann_lab/status/1554703848069111808) before we submitted the manuscript which went viral. An editor of the EMBO Journal saw this and invited us to submit our study to EMBO. This was an excellent experience and shows that a platform like Twitter can be very good for scientists, as long as it maintains its focus on science.

## This year is the 60th anniversary of the Federation of European Biochemical Societies (FEBS); how do you envision FEBS will change over the next 60 years?

Well, I hope that FEBS can help with the resolution of conflict between European and neighbouring countries. We, as scientists, have the chance to make and keep contacts across borders which are closed for politicians and others. Scientists therefore should use FEBS as a platform, for example to interact with students from countries such as Iran or Russia.

## How is  FEBS PRESS ensuring the visibility of articles in its portfolio and what are the current caveats?

I am an editor of several other journals as well. What I like about the four FEBS journals is that the editorial offices are highly professional, for example, in filtering out problematic and fraudulent manuscripts that come from paper mills before they go to peer‐review. All studies are seriously reviewed which is—as we all know—not the case for some of the new journals from other publishers. The FEBS journals also have very attractive web pages that provide a well‐illustrated overview of the content of the issues. Even if the impact factors are not as high as in some other journals, I regard them as very good and well‐respected scientific journals, at least in my community.

## Help us settle an old debate: how can we incentivise scientists to engage in peer review? What are some viable options towards their compensation?

I think we can't. Evaluating research grants and manuscripts is part of our profession. It should be an honour for every scientist to serve the community. I might be old‐fashioned, but I am convinced that trying to reward them with some Publon points or with dollars just goes in the wrong direction. The reward for active participation in these affairs is the existence of a strong and vital scientific community. This should be more than enough for encouragement.

## Conflict of interest

The authors declare no conflict of interest.

## Author contributions

IT formulated the questions, conducted the interview and edited the manuscript. JH answered the questions during the interview and provided comments on the manuscript.
